# Enhanced Incorporation of 3-Hydroxy-4-Methylvalerate Unit into Biosynthetic Polyhydroxyalkanoate Using Leucine as a Precursor

**DOI:** 10.1186/2191-0855-1-6

**Published:** 2011-05-18

**Authors:** Azusa Saika, Yoriko Watanabe, Kumar Sudesh, Hideki Abe, Takeharu Tsuge

**Affiliations:** 1 Department of Innovative and Engineered Materials, Tokyo Institute of Technology, 4259 Nagatsuta, Midori-ku, Yokohama 226-8502, Japan; 2 Ecobiomaterial Research Laboratory, School of Biological Sciences, Universiti Sains Malaysia, 11800, Penang, Malaysia; 3 Bioplastic Research Team, RIKEN Biomass Engineering Program, 2-1 Hirosawa, Wako-shi, Saitama 351-0198, Japan

**Keywords:** polyhydroxyalkanoate, copolymer, 3H4MV precursor, leucine analog resistant mutant

## Abstract

*Ralstonia eutropha *PHB^-^4 expressing *Pseudomonas *sp. 61-3 polyhydroxyalkanoate (PHA) synthase 1 (PhaC1_Ps_) synthesizes PHA copolymer containing 3-hydroxybutyrate (3HB) and a small amount (0.5 mol%) of 3-hydroxy-4-methylvalerate (3H4MV) from fructose as a carbon source. In this study, enhanced incorporation of 3H4MV into PHA was investigated using branched amino acid leucine as a precursor of 3H4MV. Leucine has the same carbon backbone as 3H4MV and is expected to be a natural and self-producible precursor. We found that the incorporation of 3H4MV was enhanced by the supplementation of excess amount (10 g/L) of leucine in the culture medium. This finding indicates that 3H4MV can be derived from leucine. To increase metabolic flux to leucine biosynthesis in the host strain by eliminating the feedback inhibition, the cells were subjected to *N*-methyl-*N'*-nitro-*N*-nitrosoguanidine (NTG) mutagenesis and leucine analog resistant mutants were generated. The mutants showed statistically higher 3H4MV fraction than the parent strain without supplementing leucine. Additionally, by supplying excess amount of leucine, the mutants synthesized 3HB-based PHA copolymer containing 3.1 mol% 3H4MV and 1.2 mol% 3-hydroxyvalerate (3HV) as minor constituents, which significantly affected the thermal properties of the copolymer. This study demonstrates that it is possible to enhance the monomer supply of 3H4MV into PHA by manipulating leucine metabolism.

## Introduction

Polyhydroxyalkanoate (PHA) is a kind of aliphatic polyester synthesized by a wide variety of microorganisms as intracellular storage and carbon source (Sudesh et al. 2000). It can be biosynthesized from renewable carbon sources such as sugars and plant oils, and can be completely biodegraded in the environment. PHA is expected to solve some environmental problems such as, excess emission of carbon dioxide, depletion of petroleum and environment pollution by waste plastics.

Poly[(*R*)-3-hydroxybutyrate], P(3HB), is the most common PHA that bacteria synthesize. However, P(3HB) is a brittle and rigid material with low flexibility because of its high crystallinity (Sudesh et al. 2000). Thus, the application of P(3HB) is limited. The mechanical properties of P(3HB) can be effectively improved by copolymerization with (*R*)-3-hydroxyalkanoate (3HA) monomers having bulky side chains such as (*R*)-3-hydroxyvalerate (3HV) (Bloembergen et al. 1986; Lee et al. 1996; Steinbüchel and Pieper 1992), (*R*)-3-hydroxyhexanoate (3HHx) (Fukui and Doi 1997; Shimamura et al. 1994; Tsuge et al. 2004) and longer 3HA (Matsusaki et al. 2000; Singh and Mallick 2009). The incorporation of such 3HA monomers lowers the crystallinity of 3HB-based copolymers due to obstacle by bulky side chain. In addition, the melting temperature of copolymer decreases with an increase in the fraction of bulky 3HA, whereas elongation at break is markedly increased (Sudesh et al. 2000). The incorporation of comonomers into P(3HB) sequence depends on the substrate specificity of the polymerizing enzyme, PHA synthase (PhaC). To date, many PhaC genes (*phaC*) have been cloned from various microorganisms and the gene products were characterized partially (Rehm 2003). In particular, the PHA synthase of *Pseudomonas *sp. 61-3 (PhaC1_Ps_) has attracted much attention because of its unique substrate specificity towards 3HA monomers with chain lengths of 4-12 carbon atoms (Matsusaki et al. 1998, 2000). Pseudomonads have several PhaCs with different substrate specificity. Since the other PhaCs from pseudomonads are unable to polymerize 3HB unit, PhaC1_Ps _has been useful for the synthesis of 3HB-based PHA copolymer incorporating various types of 3HA.

**Figure 1:**
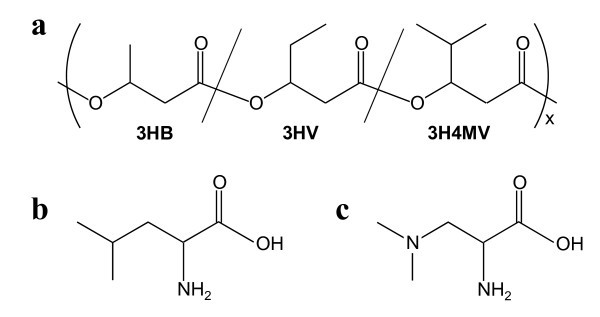
**Structures of (a) P(3HB-*co*-3HV-*co*-3H4MV), (b) leucine, and (c) 4-azaleucine (leucine analog)**.

Recently, it was shown that *Ralstonia eutropha *(currently designated *Cupriavidus necator*) strain PHB^-^4 expressing *phaC1*_Ps _has the ability to produce a new type of PHA copolymer containing branched monomer unit, termed 3-hydroxy-4-methylvalerate (3H4MV, Figure 1a), from fructose as the sole carbon source (Tanadchangsaeng et al. 2009, 2010). Both 3H4MV and 3HHx are isomers that differ only in the side chain structure, whereby 3H4MV has an iso-propyl group as the side chain whereas 3HHx has an *n*-propyl group. Therefore, P(3HB-*co*-3H4MV) and P(3HB-*co*-3HHx) copolymers showed similar mechanical and thermal properties (Tanadchangsaeng et al. 2009, 2010). The 3H4MV fraction of PHA produced from fructose by *R. eutropha *PHB^-^4 expressing *phaC1*_Ps _was only 0.5 mol%, but it can be increased up to 46 mol% by feeding 4-methylvalerate (4MV) as a 3H4MV precursor. However, since 4MV is a costly and toxic precursor, an alternative method to produce P(3HB-*co*-3H4MV) from abundant and inexpensive renewable resources is desirable.

In this study, PHA containing 3H4MV unit was synthesized by *R. eutropha *PHB^-^4 expressing *phaC1*_Ps _from fructose with or without the addition of branched amino acid, leucine, as a precursor of 3H4MV unit. Because leucine has the same carbon backbone as 3H4MV (Figure 1b), it is expected to be useful as a natural metabolite precursor of 3H4MV. In addition, mutants that are resistant to leucine analog were generated by random chemical mutagenesis and characterized for their ability to incorporate 3H4MV into PHA. This study demonstrates for the first time that it is possible to enhance the monomer supply of 3H4MV into PHA by manipulating leucine metabolism.

## Materials and methods

### Bacterial strains and plasmid

PHA-negative mutant *R. eutropha *PHB^-^4 (DSM541) was employed as host strain for PHA synthesis (Schlegel et al. 1970). The recombinant plasmid pBBR1"C1_Ps_AB_Re _containing PHA synthase gene from *Pseudomonas *sp. 61-3 (*phaC1*_Ps_) and monomer supplying enzyme genes from *R. eutropha *(*phaAB*_Re_) was transformed into the host strain by transconjugation (Tsuge et al. 2005). Leucine analog resistant mutants of *R. eutropha *PHB^-^4 were generated according to the method described below.

### Generation of leucine analog resistant mutants

*R. eutropha *PHB^-^4 expressing *phaC1*_Ps _was grown in 1.7 mL Nutrient-Rich (NR) medium (10 g of Bacto trypton, 2 g of yeast extract and 10 g of meat extract per liter of distilled water) with 50 μg/mL kanamycin at 30°C for 24 h. The cells were harvested by centrifugation and then suspended in 2.5 mL potassium phosphate buffer (100 mM, pH 7.0). Suspended cells were treated with 10 μL *N*-methyl-*N'*-nitro-*N*-nitrosoguanidine (NTG, 10 mg/mL stock solution of dimethyl sulfoxide) for 30 min at 30°C. NTG treated cells were harvested and washed three times with NR medium. Then, the cells were resuspended in NR medium and 100 μL cell suspended solution was inoculated into 1.7 mL NR medium containing 50 μg/mL kanamycin and cultivated at 30°C for 24 h. The recovered cells were spread on agar plate of mineral salt (MS) medium (9 g of Na_2_HPO_4_·12H_2_O, 1.5 g of KH_2_PO_4_, 0.5 g of NH_4_Cl, 0.2 g of MgSO_4_·7H_2_O and 1 mL of trace element solution per liter of distilled water) (Kato et al. 1996) containing 1.5 g/L 4-aza-DL-leucine dihydrochloride (Sigma Aldrich, St Louis, MO, USA, Figure 1c) as a leucine analog. After 2 days of incubation, colonies appeared on the selective agar plate which showed resistance to the leucine analog.

### HPLC assay of 3H4MV content in mutants

The 3H4MV content in leucine analog resistant mutants was measured by high-performance liquid chromatography (HPLC). Leucine analog resistant mutants were inoculated into 600 μL MS medium supplemented with 20 g/L fructose and 50 μg/mL kanamycin in 1.2 mL wells of 96 well plate. After sealing the plate with an air permeable film, the mutants were cultivated at 30°C for 72 h by shaking in a reciprocal shaker (130 strokes/min). At the end of the cultivation period, the supernatant was discarded after the mutant cells were pelleted by centrifugation. Finally, the cell pellets in the 96-well plate were dried at 55°C for 3 days.

The sample for HPLC assay was prepared by alkaline treatment, the details of which are to be published elsewhere. The method is briefly described here. The dried cell pellets were treated with 200 μL of 1N NaOH at 100°C for 3 h in a 96-well plate hermetically heat-sealed by polypropylene/aluminum film. The plate was then cooled to room temperature before adding 200 μL of 1N HCl to the cell lysate for neutralization. This sample was filtered using a 0.45 μm pore sized PTFE membrane filter plate, and the filtrates were collected into a new 96-well plate. By the alkaline treatment, the hydrolyzed 3HAs were converted to the corresponding *trans*-2-alkenoic acids.

HPLC analysis was performed using an LC-10Avp system (Shimadzu, Kyoto, Japan) with an ion-exclusion column, Fast Acid Analysis (100 mm × 7.8 mm I.D., Bio-Rad, Hercules, CA, USA), at 60°C. H_2_SO_4 _(0.014N) with 12% CH_3_CN was used as the mobile phase at a flow rate of 0.7 mL/min. The chromatograms were recorded at 210 nm by a UV detector because *trans*-2-alkenoic acids have strong UV absorption.

### PHA biosynthesis

*R. eutropha *PHB^-^4 expressing *phaC1*_Ps _and its leucine analog resistant mutants were cultured in a 500-mL shaking flask (130 strokes/min) containing 100 mL MS medium, in which nitrogen source is limited for cell growth as described above, supplemented with 20 g/L fructose at 30°C for 72 h. In all cases, 50 μg/mL kanamycin was added to the medium to maintain the plasmid stability. Five amino acids, L-leucine (L-Leu), L-valine (L-Val), L-isoleucine (L-Ile), L-threonine (L-Thr, Kanto Chemical, Tokyo, Japan) and D-leucine (D-Leu, Wako Pure Chemical, Osaka, Japan), were supplemented into MS medium to examine their ability to function as 3H4MV precursor. The cultivated cells were harvested by centrifugation and washed with distilled water to remove the medium components before being lyophilized.

### PHA analyses

PHA contents and composition were determined by gas chromatography (GC14B, Shimadzu, Kyoto, Japan) with flame ionization detector and gas chromatography-mass spectrometry (GCMS-QC 2010, Shimadzu, Kyoto, Japan). Approximately 30 mg lyophilized cells were methanolyzed in the presence of 15% sulfuric acid before analysis (Kato et al. 1996).

PHA was extracted from lyophilized cells with chloroform at room temperature, and purified by reprecipitation into methanol. Molecular weight was determined by gel permeation chromatography (10A GPC system, Shimazdu, Kyoto, Japan). Approximately 1 mg extracted polymer was dissolved in 1 mL chloroform, and analyzed at a column temperature of 40°C. Polystyrene standards with a low polydispersity were used to make the calibration curve.

PHA films for thermal analysis were prepared by solvent casting method. For this, the extracted and purified PHA was dissolved in chloroform and the polymer solution was poured into Petri dishes. The solvent was evaporated at room temperature and then the films were aged for at least three weeks to reach equilibrium crystallinity prior to analysis. For differential scanning calorimetric analysis, 2-3 mg of the PHA film was encapsulated in aluminum pans and analyzed with a Perkin-Elmer Pyris 1 DSC (Perkin-Elmer, Waltham, MA, USA) in the temperature range of -50 to 200°C at a heating rate of 20°C/min under nitrogen atmosphere.

## Results

### Effect of Amino Acid Supplementation on 3H4MV Fraction

Because the carbon back bone of 3H4MV is the same as that of branched amino acid leucine (Figure 1), we expected that leucine and its structurally related amino acids could function as 3H4MV precursors. To evaluate the feasibility of 3H4MV provision from amino acids, *R. eutropha *PHB^-^4 expressing *phaC1*_Ps _was cultivated in MS plus fructose medium supplemented with 10 g/L of various amino acids. Table 1 shows the result of cultivation. The dry cell weights increased with the addition of amino acids except for L-valine and D-leucine. L-Valine has been used for PHA production (Fujita et al. 1993, Kimura et al. 2003); however, effect of high concentration of L-valine (10 g/L) on the cell growth has not been reported previously. L-Isoleucine is known to function as a 3HV precursor in *R. eutropha *(Steinbüchel and Pieper 1992). Our result also showed that the addition of L-isoleucine enhanced the 3HV fraction to 7.7 mol%. The same effect was also demonstrated by L-threonine (Steinbüchel and Pieper 1992), but no enhancement of 3HV was seen in our study. As for 3H4MV, a very small amount of 3H4MV (0.5 mol%) was incorporated into PHA when no amino acids were supplemented. Supplementation of L-isoleucine and L-threonine also showed no effect on 3H4MV enhancement. However, L-leucine supplementation showed a slightly increased 3H4MV fraction (0.9 mol%), suggesting that L-leucine (hereinafter referred to as leucine) is a potent candidate of 3H4MV precursor.

**Table 1:** PHA biosynthesis by *R. eutropha *PHB^-^4 expressing *phaC1*_Ps _with the supplementation of various amino acids

	Dry cell weight (g/L)	PHA content (wt%)	**PHA composition (mol%) **^**a**^		
			
Amino acid			3HB	3HV	3H4MV
none	1.6	53	99.1	0.4	0.5
L-Val	trace ^b^	-	-	-	-
L-Leu	7.2	29	98.8	0.3	0.9
L-Ile	5.7	17	92.3	7.7	0
L-Thr	7.6	43	99.3	0.4	0.3
D-Leu	trace ^b^	-	-	-	-

Cells were cultured in MS plus fructose (20 g/L) medium supplemented with each amino acid (10 g/L). The results are the average of three independent cultivations (the standard deviations were less than 5% of the mean).

^a ^PHA composition was determined by GC.

^b ^less than 0.1 g/L.

### PHA Production by Leucine Analog Resistant Mutants

From the result of leucine supplementation, it was speculated that 3H4MV provision might be increased by increasing the metabolic flux to leucine biosynthesis, without the use of 3H4MV precursor. However, leucine biosynthesis pathway is known to be strictly regulated by end product feedback inhibition. To eliminate the feedback inhibition, we aimed to generate leucine analog resistant mutants of *R. eutropha *PHB^-^4 harboring *phaC1*_Ps _by NTG mutagenesis, using the same approach that was used for the generation of L-leucine producers of *E. coli*. (Nakano et al. 1996)

**Figure 2:**
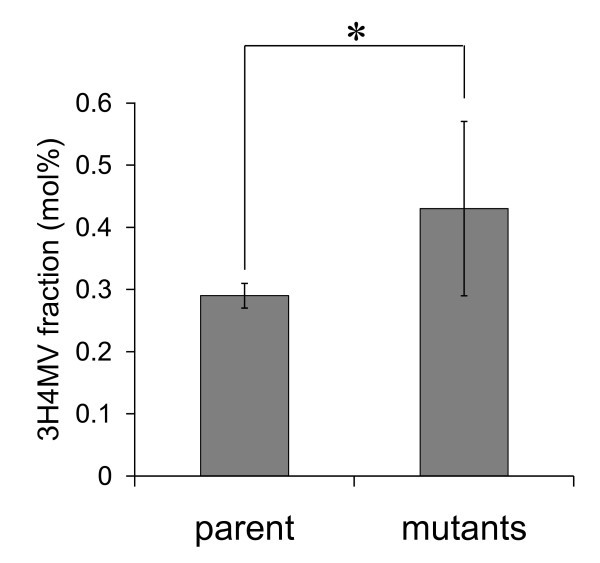
**Average 3H4MV fraction in PHA synthesized by *R. eutropha *PHB**^-^**4 expressing *phaC1***_**Ps **_**(parent strain) and leucine analog resistant mutants (mutant strains) using MS plus fructose (20 g/L) medium**. Number of repeated culture of the parent strain and number of analyzed colonies were 20 and 440, respectively. 3H4MV fractions were determined by HPLC analysis. Statistical analyses were performed with Student's t-test (*P < 0.01).

More than a thousand leucine analog resistant mutants of *R. eutropha *PHB^-^4 harboring *phaC1*_Ps _were generated by the mutagenesis. These mutants were cultured in 96-deep well plate with MS medium plus fructose as a sole carbon source to analyze the PHA composition by high-throughput HPLC. As a result, 440 leucine analog resistant mutants accumulated detectable amount of PHA. Figure 2 shows the comparison of average 3H4MV fractions between *R. eutropha *PHB^-^4 expressing *phaC1*_Ps _(parent strain) and leucine analog resistant mutants. The average 3H4MV fraction of the parent strain was 0.29 mol% (number of repeated culture, n = 20) in this assay condition, whereas that of leucine analog resistant mutants showed 0.43 mol% (number of analyzed colonies, n = 440), which showed a statistically significant increase in 3H4MV fraction. The impaired leucine feedback system of these mutants resulted in increased 3H4MV fraction due to the increased metabolic flux to leucine biosynthesis.

Four leucine analog resistant mutants showing significantly higher 3H4MV fraction, designated as 1F2, 6C1, 12D1 and 13H3, were selected for further characterization. These mutants were cultivated in shaken flasks containing 100 mL MS plus fructose medium for 72 h at 30°C. Table 2 shows the result of cultivation and PHA composition determined by GC. These mutants showed approximately 2-fold higher 3H4MV fraction (up to 0.9 mol%) than the parent strain (0.5 mol%). As for 3HV unit, the mutants (1.5-1.7 mol%) showed up to 4-fold higher fraction than the parent strain (0.4 mol%). There was no significant effect on cell growth and PHA content among the four mutants and the parent strain.

**Table 2:** PHA biosynthesis by *R. eutropha *PHB^-^4 expressing *phaC1*_Ps _or the leucine analog resistant mutants from fructose as the sole carbon source

	Dry cell weight (g/L)	PHA content (wt%)	**PHA composition (mol%) **^**a**^		
			
Strain			3HB	3HV	3H4MV
Parent ^b^	1.6	53	99.1	0.4	0.5
1F2 ^c^	1.5	53	97.6	1.6	0.8
6C1 ^c^	1.7	55	97.6	1.5	0.9
12D1 ^c^	1.6	51	97.4	1.7	0.9
13H3 ^c^	1.7	51	97.4	1.7	0.9

Cells were cultured in MS plus fructose (20 g/L) medium. The results are the averages of three independent cultivations (the standard deviations were less than 5% of the mean).

^a ^PHA composition was determined by GC.

^b ^*R. eutropha *PHB^-^4 expressing *phaC1*_Ps._

^c ^leucine analog resistant mutants.

### PHA Production by Mutants with Leucine Supplementation

The maximum 3H4MV fraction achieved so far was less than 1 mol% even by using leucine analog resistant mutants or feeding leucine as a 3H4MV precursor. To further increase the 3H4MV fraction, the above four mutants were cultured in MS plus fructose medium supplemented with excess amount of leucine (10 g/L). Table 3 shows the result of cultivation. The parent strain showed 0.9 mol% 3H4MV fraction, whereas the mutants showed significantly increased 3H4MV fraction in the range of 2.5-3.0 mol%. Also, 3HV fractions were also increased to 1.0-1.4 mol%. The cell growth of the mutants was at the same level as the parent strain, but the PHA content was slightly increased. The combination of leucine analog resistant mutant and leucine supplementation was effective to increase 3H4MV fraction.

**Table 3:** PHA biosynthesis by *R. eutropha *PHB^-^4 expressing *phaC1*_Ps _or leucine analog resistant mutants with the supplementation of 10 g/L leucine

	Dry cell weight (g/L)	PHA content (wt%)	**PHA composition (mol%) **^**a**^		
			
Strain			3HB	3HV	3H4MV
Parent ^b^	7.2	29	98.8	0.3	0.9
1F2 ^c^	7.1	34	95.8	1.2	3.0
6C1 ^c^	7.2	36	96.5	1.0	2.5
12D1 ^c^	7.3	35	95.8	1.4	2.8
13H3 ^c^	7.2	35	95.9	1.3	2.8

Cells were cultured in MS plus fructose (20 g/L) medium supplemented with L-leucine (10 g/L). The results are the averages of three independent cultivations (the standard deviations were less than 5% of the mean).

^a ^PHA composition was determined by GC.

^b ^*R. eutropha *PHB^-^4 expressing *phaC1*_Ps._

^c ^leucine analog resistant mutants.

**Figure 3:**
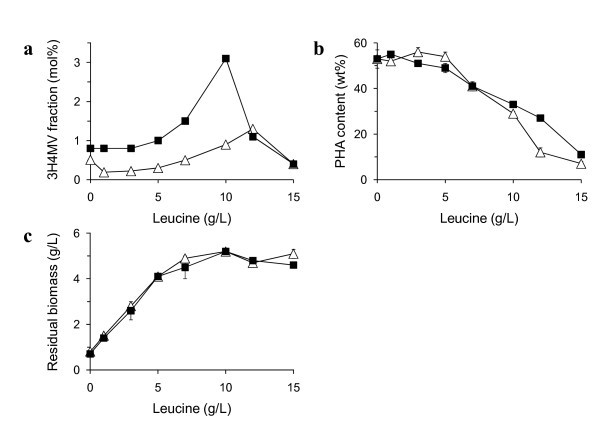
**Cultivation of *R. eutropha *PHB**^-^**4 expressing *phaC1***_**Ps **_**(open triangle) and the leucine analog resistant strain 1F2 (closed square) in the presence of various concentration of L-leucine (0-15 g/L) and fructose (20 g/L)**. (a) 3H4MV fraction in PHA copolymers, (b) PHA contents in the cells, (c) residual biomass (obtained by subtracting PHA weight from dry cell weight).

To examine the relationship between 3H4MV fraction and leucine concentration in the medium, the parent strain and the mutant 1F2 were cultivated using various concentrations of leucine. The 3H4MV fractions in PHA are compared in Figure 3a. Both strains showed an increase in 3H4MV fraction with increasing leucine concentration from 5 to 10 g/L. The 3H4MV fraction in the mutant 1F2 reached 3 mol% at 10 g/L leucine, whereas the parent strain showed the maximum 3H4MV fraction at 12 g/L leucine. Figures. 3b and 3c show the PHA content and residual biomass of both strains, respectively. The PHA contents decreased with increasing leucine concentration due to the sufficient supply of nitrogen source. It is well known that PHA synthesis is repressed under nitrogen-rich condition (Sudesh et al, 2000). In contrast, production of residual biomass was prompted by excess amount of nitrogen derived from leucine. At the leucine concentration of up to 5 g/L, leucine was preferentially used for residual biomass production (Figure 3c). When the leucine concentration was more than 5 g/L, the residual biomass reached a plateau probably due to the shortage of some nutrition other than nitrogen source. Therefore, the excess leucine would be converted to 3H4MV, instead of residual biomass, at 5-12 g/L of leucine concentration.

### Characterization of PHA Synthesized by Mutant 1F2

Molecular weights and thermal properties of PHA synthesized by mutant 1F2 in the presence of leucine were characterized. The 3H4MV fractions were varied by changing leucine concentrations in the medium. Table 4 shows the molecular weights and thermal properties of the resulting PHA. The number average molecular weight (*M*_*n*_) and the weight average molecular weight (*M*_*w*_) decreased from 251 × 10^3 ^to 98 × 10^3 ^and 479 × 10^3 ^to 160 × 10^3^, respectively, as leucine concentration increased from 0 to 10 g/L. The polydispersity indexes (*M*_*w*_/*M*_*n*_) were in the range of 1.6-1.9. As the 3HV plus 3H4MV fractions increased from 0 to 4.3 mol%, melting temperature (*T*_m_) decreased drastically from 172°C to 137°C (lower *T*_m_) and 151°C (higher *T*_m_). The enthalpy of fusion (Δ*H*_m_), which relates to degree of crystallinity, was also decreased. Meanwhile, the glass-transition temperature (*T*_g_) showed little change. It was revealed that small amounts of 3HV and 3H4MV affected the *T*_m _and the Δ*H*_m _of the PHA copolymers to a great extent.

**Table 4:** Thermal properties of PHA containing 3H4MV synthesized by the mutant 1F2 using leucine as a 3H4MV precursor, P(3HB-*co*-3HV), and P(3HB-*co*-3HHx)

		**PHA composition **^**a**^				Thermal property			Molecular weight	
				
Polymer	Leucine (g/L)	3HV (mol%)	3H4MV (mol%)	3HHx (mol%)	**Total **^**b **^**(mol%)**	***T***_**m **_**(°C)**	***T***_**g **_**(°C)**	**Δ*H***_**m **_**(J/g)**	***M***_**n **_**(×10**^**3**^**)**	*M*_w_/*M*_n_
P(3HB-*co*-3HV-*co*-3H4MV)^c^	0	1.6	0.8	0	2.4	146, 159	3	42	250	1.9
P(3HB-*co*-3HV-*co*-3H4MV)^c^	5	2.3	1.0	0	3.3	142, 154	3	40	251	1.8
P(3HB-*co*-3HV-*co*-3H4MV)^c^	10	1.2	3.1	0	4.3	137, 151	3	42	98	1.6
P(3HB) ^d^	-	0	0	0	0	172	4	77	224	2.1
P(3HB-*co*-3HV) ^e^	-	8	0	0	8	170	-	70	-	-
P(3HB-*co*-3HHx) ^f^	-	0	0	5	5	151	0	69	100	1.9

*M*_n_, number-average molecular weight; *M*_w_, weight-average molecular weight; *M*_w_/*M*_n_; polydispersity index; *T*_m_, melting temperature; *T*_g_, glass-transition temperature; Δ*H*_m_, enthalpy of fusion.

^a ^PHA compositions of purified copolymer samples were determined by GC. Copolymer compositions other than 3HB are shown.

^b ^3HV plus 3H4MV plus 3HHx fraction.

^c ^PHA synthesized by mutant 1F2 from fructose (20 g/L) and leucine (0, 5, 10 g/L).

^d ^P(3HB) homopolymer synthesized by *R. eutropha *H16.

^e ^(Scandola et al. 1992).

^f ^(Doi et al. 1995).

## Discussion

Previous studies showed that 3H4MV unit which has iso-propyl side chain was incorporated into PHA from fructose as the sole carbon source (Tanadchangsaeng et al. 2009). However, the 3H4MV fraction was too low (0.5 mol%) to improve the properties of 3HB-based polymer. Thus we attempted to increase the 3H4MV fraction by using 3H4MV precursors. (Tanadchangsaeng et al. 2009) showed that 4-methylvalerate and 4-methyl-2-pentenoate, which are branched fatty acids structurally similar to 3H4MV, were able to increase 3H4MV fraction effectively. However, these precursors are not only costly but they also significantly inhibit bacterial cell growth. Therefore, we have sought a novel precursor able to be produced as a natural metabolite in bacterial cells such as branched amino acids.

There have been many reports on the use of amino acids to increase second monomer unit, especially 3HV unit, in 3HB-based PHA copolymer. It is known that isoleucine, threonine and valine are effective in increasing 3HV unit (Fujita et al. 1993; Kimura et al. 2003; Nakamura et al. 1992). These amino acids are partially converted to propionyl-CoA which is an intermediate of 3HV biosynthesis pathway in the cells (Steinbüchel and Pieper 1991). (Choi et al. 2003) demonstrated that the threonine-overproducing mutant of *Alcaligenes *sp. SH-69 synthesized P(3HB-*co*-3HV) with 3HV fraction of up to 22 mol% (3-fold higher than the parent strain) from glucose as the sole carbon source, without external amino acid supplementation. As seen from above, the amino acids have been widely used as 3HV precursors for P(3HB-*co*-3HV) synthesis. In contrast, there are no reports of P(3HB-*co*-3H4MV) synthesis by using amino acids as a 3H4MV precursor. (Tanadchangsaeng et al. 2009) showed that supplementation of 1 g/L leucine had negative effect on 3H4MV fraction. In this study, we also observed the negative effect on 3H4MV fraction at low concentration of leucine (1-5 g/L) in the parent strain (Figure 3a). However, supplementation of excess leucine (10-12 g/L) resulted in increased 3H4MV fraction (Table 1 and Figure 3a), suggesting that 3H4MV unit can be derived from leucine.

Our results showed that leucine analog resistant mutant of *R. eutropha *was able to increase the 3H4MV fraction even when fructose was used as the sole carbon source (Figure 2 and Table 2). The leucine analog resistant *E. coli *has been employed to produce leucine as an extracellular product. The high leucine productivity of 3.4 g/L was achieved by the *E. coli *mutants that are tolerable to 1 g/L of leucine analog (4-azaleucine, Nakano et al. 1996). Unlike *E. coli *mutant, the four *R. eutropha *mutants generated in this study (1F2, 6C1, 12D1 and 13H3) did not secrete leucine to the culture medium, as revealed by HPLC analysis (data not shown). However, these mutants showed good growth even in the presence of 3 g/L leucine analog. This concentration is 2-fold higher than that used for the screening for leucine analog resistant mutants. In general, the mutants that were able to grow in high concentration of leucine analog have an impaired feedback system in leucine biosynthesis pathway, resulting in the overproduction of leucine. Therefore, the increased 3H4MV in the mutants observed here could be attributed to increased leucine production in the cells.

We presumed that the major difference between the parent strain and the four leucine analog resistant mutants (1F2, 6C1, 12D1 and 13H3) is in the leucine biosynthesis pathway with or without feedback system. However, leucine supplementation (10 g/L) to these cultures resulted in significantly different 3H4MV fraction (Figure 3 and Table 3). This difference could not be explained by the leucine feedback system only. To eliminate the effect of mutation in the plasmid, we performed plasmid curing of the resistant mutant 1F2 and then pBBR1"C1_Ps_AB_Re _plasmid was transformed again. The mutant harboring fresh plasmid showed the same dry cell weight, PHA content and PHA composition as the original strain (data not shown). Because the above four mutants were selected from leucine analog resistant library by HPLC assay based on 3H4MV fraction, they might have other mutations specifically in the 3H4MV biosynthesis-related genes. Since 3H4MV biosynthesis pathway has not yet been identified, these mutants might be useful in the study of this pathway.

PHA copolymers that were synthesized by the mutant 1F2 with leucine supplementation showed low melting temperatures, depending on 3H4MV and 3HV fractions (Table 4). P(3HB-*co*-3HV) is the most popular 3HB-based copolymer, however, the incorporation of 8 mol% 3HV unit into P(3HB) sequences did not influence the melting temperature (Scandola et al. 1992). Meanwhile, only 5 mol% of 3HHx was enough to decrease the melting temperature by 20°C (Doi et al. 1995). In this study, 4.3 mol% of 3H4MV and 3HV fractions had the same effect as 3HHx for decreasing the melting temperature by 20°C. The effect of 3H4MV on melting temperature was also demonstrated by the PHA copolymers synthesized by other types of bacteria (Chia et al, 2010; Lau et al 2010, 2011). In the hot melt processing of P(3HB) materials, one of the major problems is the decrease in molecular weight of polymers due to rapid thermal degradation near its melting temperature. Reducing the melting temperature of the polymer allows for lower processing temperatures in the hot melt processing, without decreasing molecular weight. Therefore, 3HB-based copolymer containing small amount of 3H4MV and 3HV fractions would be practical in terms of not only mechanical properties but also thermal properties.

In conclusion, this study demonstrated that 3H4MV fraction in PHA can be increased by feeding excess leucine as a precursor of 3H4MV unit or employing the leucine analog resistant mutants. Moreover, by combining these two factors, 3H4MV fraction was increased up to 3.1 mol%. This study is the first step in establishing the P(3HB-*co*-3H4MV) biosynthesis from unrelated carbon sources such as sugars as the sole carbon source by focusing on the leucine metabolism.

## Competing interests

The authors declare that they have no competing interests.
